# Children show right-lateralized effects of spoken word-form learning

**DOI:** 10.1371/journal.pone.0171034

**Published:** 2017-02-03

**Authors:** Anni Nora, Leena Karvonen, Hanna Renvall, Tiina Parviainen, Jeong-Young Kim, Elisabet Service, Riitta Salmelin

**Affiliations:** 1 Department of Neuroscience and Biomedical Engineering, and Aalto NeuroImaging, Aalto University, Espoo, Finland; 2 Department of Psychology, University of Jyväskylä, Jyväskylä, Finland; 3 Department of World Cultures, University of Helsinki, Helsinki, Finland; 4 Department of Linguistics and Languages, McMaster University, Hamilton, Canada; Utrecht University, NETHERLANDS

## Abstract

It is commonly thought that phonological learning is different in young children compared to adults, possibly due to the speech processing system not yet having reached full native-language specialization. However, the neurocognitive mechanisms of phonological learning in children are poorly understood. We employed magnetoencephalography (MEG) to track cortical correlates of incidental learning of meaningless word forms over two days as 6–8-year-olds overtly repeated them. Native (Finnish) pseudowords were compared with words of foreign sound structure (Korean) to investigate whether the cortical learning effects would be more dependent on previous proficiency in the language rather than maturational factors. Half of the items were encountered four times on the first day and once more on the following day. Incidental learning of these recurring word forms manifested as improved repetition accuracy and a correlated reduction of activation in the right superior temporal cortex, similarly for both languages and on both experimental days, and in contrast to a salient left-hemisphere emphasis previously reported in adults. We propose that children, when learning new word forms in either native or foreign language, are not yet constrained by left-hemispheric segmental processing and established sublexical native-language representations. Instead, they may rely more on supra-segmental contours and prosody.

## Introduction

It is often stated that children learn a new language differently from adults, just by exposure and imitation. Indeed, the ability to form memory representations for meaningless word forms and reproduce them has been shown to be important especially for the early stages of vocabulary learning [1, 2]. The neural underpinnings of incidental phonological learning through exposure and overt repetition have been recently studied in adults [3–6]. Despite abundant research on the neural-level processes of phonological learning also in young infants [7–10], there are very few studies investigating older, school-aged children. Knowledge of the development of the neural architecture for phonological processing and learning is crucial for understanding possible changes in language acquisition potential, and may also have significant implications for populations struggling with the sound system of a language.

It is well documented that the ability to learn to perceive phoneme contrasts outside one’s native language decreases during development, reflecting the influence of linguistic environment on the speech processing system [11]. Native phonotactic regularities, prosodic features, as well as lexical phonological representations also begin to affect spoken word form processing and learning during the first year of life [12, 13]. However, phonological processing undergoes changes well into school age [14], and it is at this age when more explicit phonemic parsing and phonological awareness fully arise, in interaction with reading skills [15, 16]. The gradually developing specialization for native language might have an effect on how foreign phonology is processed and acquired at different ages. Studies on immigrants have consistently found age-of-acquisition effects on second-language learning, particularly on attainment of the sound structure and native-like pronunciation [17, 18]. In contrast, controlled behavioral studies comparing phonological learning in adults and children have found no differences in learning new foreign word forms [19] or artificial phonotactic regularities [20, 21] within session. Yet, cortical activation might be modified without any salient differences in performance within session [22], while behavioral benefits for children could surface over a delay or with extended exposure [23, 24].

Neuroimaging studies have revealed striking differences in the overall cortical sequence of auditory processing in children compared to adults. Auditory evoked responses undergo many changes from childhood to adulthood, with mature features appearing somewhat earlier in the right than left hemisphere [25–28]. These responses reflect the state of the developing speech processing system and are linked to linguistic skills [25, 29]. Infants and children show prominent bilateral cortical activation during linguistic processing, and the development of leftward lateralization of language processing continues into school age [30–34].

The reorganization of the speech processing system due to native language exposure may also be reflected in cortical learning effects. Cortical familiarity effects at 200–400 ms after stimulus onset have been observed in infants for known or newly-learned words paired with referents in comparison with unknown words, initially bilaterally but becoming more left-lateralized with increasing proficiency in the language [8, 9, 35]. In these studies, however, the effects of phonological word form familiarity are indistinguishable from effects of lexical-semantic familiarity. Most of these studies have used native language pseudowords as stimuli. Understanding the contribution of the developing native language specialization in phonological learning would require comparison between brain responses to new word forms that either are or are not part of the native language sound system.

The present study addresses learning of new meaningless foreign and native-like word forms in a narrow age group of 6–8-year-old Finnish-speaking children. We used an incidental learning task with overt repetition and repeated exposure to meaningless word forms, developed in our earlier studies on adults [3, 36] as well as children [19]. We first addressed repetition accuracy and explicit recognition memory for novel foreign (Korean) words in a behavioral experiment. Half of the stimuli were presented four times during a session and again on the following day. In another group of children, we tracked the cortical correlates of learning with magnetoencephalography (MEG) while the participants repeated four-syllable foreign (Korean) words and native (Finnish) pseudowords. Based on an earlier behavioral study [19], we expected to see improvement in repetition of recurring word forms that is comparable to adults. Cortical learning effects were expected to be confined to sustained responses (> 300 ms after word onset), comparably to what has been shown for adults and infants. Possible dissimilarities to previous results on adults [3, 36] and the direct comparison of learning native vs. foreign word forms may provide insight into how the developing specialization for native language affects the cortical organization of phonological processing and learning.

## Materials and methods

### Participants

Fifteen children (9 females and 6 males) mean age 7 years 9 months (ranging from 6 years 7 months to 8 years 8 months) participated in the *Behavioral experiment*. In the *MEG experiment*, data were collected from another group of 13 children (7 females and 6 males) with mean age of 7 years 2 months (ranging from 6 years 3 months to 7 years 10 months). Participants in both experiments were Finnish-speaking monolinguals. Three children participating in the *Behavioral experiment* had some experience with foreign languages (not Korean or related languages) in school or daycare. Four additional children participated in MEG measurements, but were excluded from analysis because of large motor artifacts or failure to complete all brain imaging sessions. All participants were dominant native speakers of Finnish. Participants were right-handed as established with an adapted version of the Edinburgh Handedness Inventory [37]. All had normal hearing and no history of neurological or language disorders. Possible phonological working memory deficits were screened in both experiments with the WISC-R/WAIS-R Digit Span subtest and a pseudoword repetition task. The children’s families were contacted through elementary schools, one after-school club and one summer camp. This study was approved by the Helsinki University Central Hospital Coordinating Ethics Committee (*MEG experiment*) and Aalto University Research Ethics Committee (*Behavioral experiment*). The children’s parents gave their written informed consent. Children’s oral consent was also obtained. The children received a small gift as a thank you for participating.

### Stimuli

The stimuli consisted of a set of 320 four-syllable foreign (Korean) words and 320 four-syllable native (Finnish) pseudowords [3]. Korean and Finnish have different phonemic systems and they differ considerably in their typical syllable structure; native Finnish speakers experience difficulty perceiving and producing unfamiliar Korean phoneme contrasts. Both languages have primarily word-initial stress patterns. The Korean words were selected and digitally recorded by a female native Korean linguist, speaking standard Korean. The Finnish pseudoword stimuli were four-syllable words or composed of pairs of two-syllable words no longer in use, selected by the authors from an old dictionary [38] and recorded by a female native Finnish speech pathology student. Words in both languages were mostly nouns. The duration of the words varied from 0.85 to 1.46 s (mean 1.14 s) for Korean and from 1.01 to 1.49 s (mean 1.26 s) for Finnish items. All stimuli began with a consonant, and stimuli in both languages contained equal numbers of nasal, fricative/affricate, and stop consonants as initial consonants in each stimulus category.

The words were recorded in 24-bit wav format using a sampling rate of 48 kHz and, to suppress background noise, low-passed at ~6 kHz (gradual slope from 4 kHz to 14 kHz). A 10-ms ramp was added to the beginning and end of each word. Stimuli were equalized, as much as possible, with respect to acoustic properties that are known to have a strong influence on early auditory cortical responses, such as stimulus intensity, length, and rise time [39, 40]; however, some natural differences between the two languages and the two different speakers necessarily remained.

### Design and procedure

The experimental paradigm in both the *Behavioral* and the *MEG experiment* was nearly identical to our previous study on adults [3]. However, in order to reduce the experiment duration for children, the paradigm was modified slightly: The recurring word forms were presented four times (instead of five times as in the original study) and there were fewer stimuli per stimulus type (80 instead of 100). The participants’ task was to listen to the words through headphones (in the *Behavioral experiment*) or a panel speaker (in the *MEG experiment*) with 75 dB gain, and to repeat them as accurately as possible. Participants were not instructed to memorize the words. In the *Behavioral experiment*, participants repeated each word form immediately following presentation. The presentation rate of the stimuli was controlled by the experimenter. The *MEG experiment* employed delayed repetition to avoid muscle artifact contamination in the MEG signal. After each stimulus presentation and 300 ms of silence, a 50-ms beep prompted the participant to overtly repeat the item. Participants had 2 s to repeat the heard word form. In both experiments, the participants’ responses were recorded using a digital recorder and later evaluated for accuracy.

**Fig 1:**
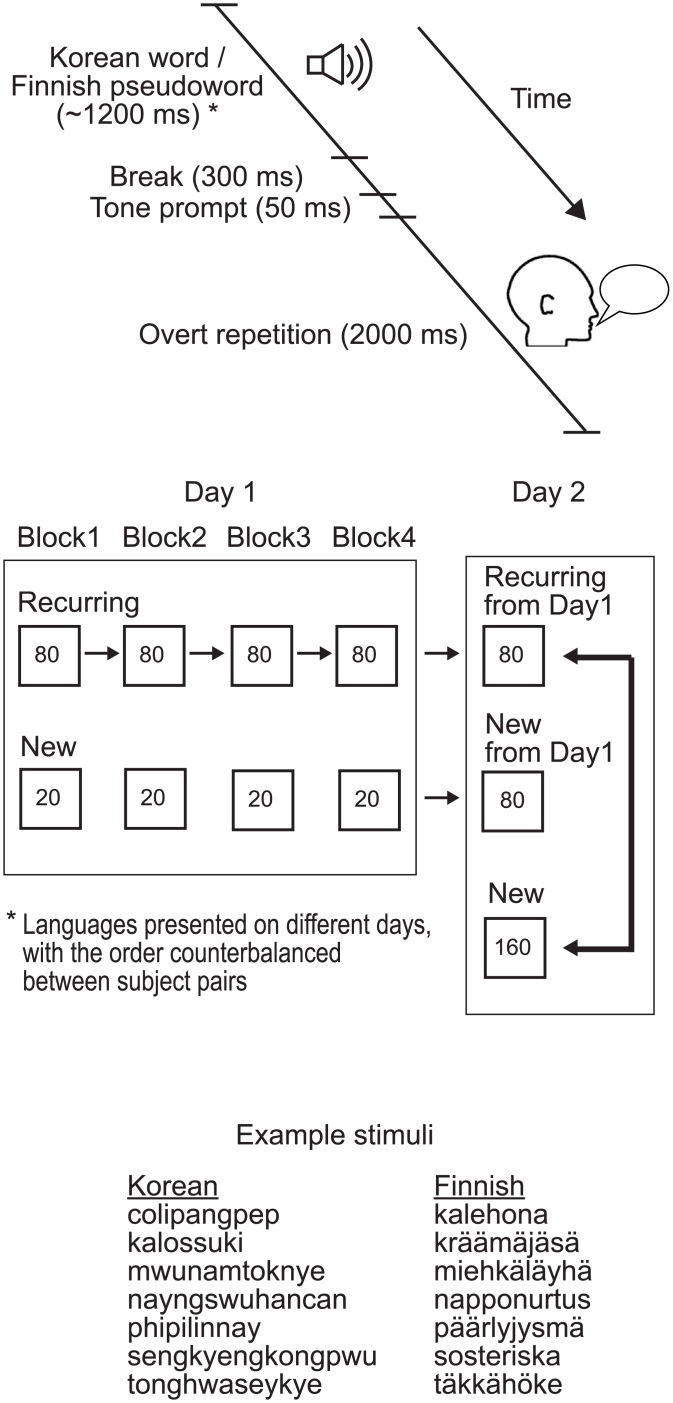
MEG experimental design. Subjects listened to and overtly reproduced foreign (Korean) words and native (Finnish) pseudowords, each language presented on two consecutive days. No explicit instructions to memorize the words were given. On Day 1 for each language, subjects heard 80 Recurring items (4 times) and 80 New items (20 in each of the four blocks). On the consecutive Day 2, the same Recurring stimuli (80) were presented once, randomly mixed with 160 completely New word forms (a subset of 80 was used in the analysis) and one presentation of the stimuli (80) that had been presented as new stimuli during Day 1 (80; their single presentation on Day 2 was not used in the analysis). At the bottom are examples of Korean words (in romanized form) and Finnish pseudowords used as stimuli.

On Day 1 of both the *Behavioral* and the *MEG experiment*, half of the stimuli (80) were presented four times (“Recurring”) and half (80) only once (“New”) (Fig 1). The stimuli were presented in four blocks, each containing one presentation of the Recurring items (80), intermixed with 1/4 (20) of the New items of Day 1. On the consecutive Day 2, the same Recurring stimuli (80) were presented once more (the fifth time), randomly mixed with 160 completely New word forms and one presentation of the stimuli that had been presented as New stimuli during Day 1 (80; this second repetition of Day 1 New stimuli on Day 2 was not included in the analysis).

The *Behavioral experiment* was conducted in the foreign language only, on two consecutive days. The testing lasted approximately 30 minutes on Day 1 and 25 minutes on Day 2, with 5 short breaks during both sessions. The *MEG experiment* was conducted on two consecutive days for each language, in separate sessions; thus, there were altogether 4 days of MEG recordings per participant. The order of the languages was counterbalanced between participants, and 2 to 8 weeks passed between measurements in the different languages. The MEG recording lasted approximately 25 minutes on Day 1 and 20 minutes on Day 2, with two short breaks inside and one longer break outside the shielded room on both days.

On Day 2 of the *Behavioral experiment*, the word-repetition task was followed by a recognition task that the participants had no advance knowledge of. Participants heard through headphones a randomly selected subset of foreign words from Day 1 (20 of which were Recurring and 20 that had been presented only once during Day 1) mixed with a matching number (40) of completely New words they had not heard before. The participants' task was to indicate by a button press for each word if it had been presented before.

### MEG recording

Magnetic fields associated with neural current flow were recorded with a 306-channel whole-head neuromagnetometer (Elekta Oy) in a magnetically shielded room at the Aalto NeuroImaging MEG Core. The MEG signals were band-pass filtered between 0.03 and 200 Hz and sampled at 600 Hz. Eye movements and blink artifacts were monitored by electro-oculogram (EOG) and motor artifacts related to mouth movements by electro-myogram (EMG), each measured with two electrodes that were placed diagonally around the eyes and the mouth, respectively. The position of the participant’s head within the MEG helmet was defined using five head position indicator coils, attached to the participant’s scalp. The locations of these coils were determined with respect to three anatomical landmarks (nasion and two preauricular reference points) with a 3D digitizer and to the sensor array by briefly feeding current to the coils. Head movements were monitored continuously [41].

### Anatomical magnetic resonance imaging

Anatomical magnetic resonance images (MRIs) were obtained with a 3T MRI scanner (Magnetom Skyra, Siemens) for all children who participated in the study. The scan included a 3-plane localizer and a T1-weighted anatomical image. The MEG data were co-registered in the same coordinate system with the individual anatomical MR images to allow attribution of MEG activation patterns to cortical loci.

### Behavioral analysis

The overt repetitions produced by the participants in the *Behavioral experiment* were rated in a scrambled order by two native Korean speakers. The responses included repetitions from the 1^st^ and 4^th^ block of Day 1 (80 Recurring + 20 New stimuli for both blocks), and from Day 2 a randomly selected subset of stimuli from the middle of the session (containing approximately 20 Recurring and 40 New word forms). Each rater gave one point per word if all the phonemes in the word could be perceived, i.e. if none were omitted, replaced or transposed; otherwise zero points. Phonetically native-like articulation or prosody was, however, not required, following the practice in foreign word repetition research [21, 42]. As there is some degree of subjectivity in the ratings, the results of the two raters were averaged (inter-rater reliability: Cronbach’s alpha 0.752). Recognition performance was evaluated as hits to previously heard (Recurring) words, false alarms to New words (presented for the first time in the recognition task), and a discriminability measure *d’* between these two, calculated individually for each participant.

In the *MEG experiment*, due to technical problems audio recordings were only available for 8 participants and not for all conditions for all participants. The repetitions of Recurring and New foreign and native word forms were rated in a scrambled order by a single native speaker of Korean and a speaker of Finnish, respectively, using word level scoring.

To offer a behavioral baseline in adults for comparison with the present data on children, adult Korean repetition data collected during a previous experiment with the same paradigm, on two consecutive days [3], was rated by a single native speaker of Korean, and only word level scoring was used.

### MEG data analysis

The MEG signal analysis focused on the perception phase before overt production. Spatio-temporal signal space separation (tSSS [43]), and movement compensation algorithms [41] were applied offline to the raw data using Max-Filter^™^ software (Elekta Neuromag) to remove the effects of external interference and to compensate for head movements during the measurement. To obtain an estimate of the artifact signals caused by blinks or saccades, the MEG signals were averaged with respect to the EOG signal. Principal component analysis (PCA) was performed on this average, and the magnetic field component produced by the eye movements was removed from the raw data [44]. Similar artifact removal was done for cardiac artefacts, for which averaging was done based on thresholding the MEG signal in channels containing the most salient cardiac response.

The resulting signals were averaged from 200 ms before to 1200 ms after the stimulus onset, rejecting trials contaminated by large artifacts (signal strength exceeding 3000 fT/cm). On average 79 ± 0.3 (mean ± standard deviation) artifact-free epochs per subject were gathered for each of the categories (maximum = 80). The averaged MEG responses were baseline-corrected to the 200-ms interval immediately preceding the stimulus onset and low-pass filtered at 40 Hz.

An overview of the underlying sources of neural activity was obtained with minimum norm estimates (MNE [45]) using the MNE Suite software package [46]. The cortical surface of each subject was reconstructed from the corresponding magnetic resonance (MR) images with the Freesurfer software [47, 48]. Each hemisphere was covered with ~5000 potential source locations. Currents oriented normal to the cortical surface were favored by weighting the transverse currents by a factor of 0.2, and depth-weighting was used to reduce the bias towards superficial sources [49]. Noise-normalized MNEs (dynamical Statistical Parametric Maps, dSPMs) were computed over the whole cortical area to estimate the signal-to-noise ratios in each potential source location [50]. The noise covariance matrix was estimated from the 200-ms prestimulus baseline periods in the raw data. For group-level visualization, the MNEs of individual subjects were first normalized to the maximum value of that subject across all conditions and subsequently morphed, with spatial smoothing, to fsaverage template.

Next, separable cortical-level spatiotemporal components were estimated by means of guided current modeling (Equivalent Current Dipole modeling, ECD [51]). ECD analysis can distinguish between multiple spatially close neural sources with different orientations of current flow. Only ECDs explaining more than 80% of the local field variance were accepted in the model. This criterion led to the inclusion of 3–5 ECD components per participant. For any one participant, the ECDs represented well the data of all recording sessions. The early transient and late sustained activations could not be captured well by a single source model, and thus were modeled separately. Sources in bilateral superior temporal and left frontal areas were found in at least 9 of the 13 children, and were included in group-level analysis. The time courses of the identified spatiotemporal components (source waveforms) were estimated by fixing their location and orientation parameters while allowing their strengths to vary to best account for the signals detected by all MEG sensors over the entire analysis interval. To locate the ECD components anatomically, the center of activation of each component was displayed on the individual magnetic resonance images (MRIs) of each participant. For group-level visualization, the locations were transformed to fsaverage surface template.

### Statistical analysis

To analyze repetition accuracy of the Korean words (in both experiments), the Recurring stimuli (= the 1^st^ and 4^th^ presentations of Recurring words from Day 1 and their single 5^th^ presentation from Day 2) and the New stimuli (= Nonrecurring stimuli from 1^st^ and 4^th^ block on Day 1 and New stimuli from Day 2) were included in a repeated-measures 2 x 3 ANOVA with the within-subjects factors Stimulus type (Recurring vs. New) and Time of testing (1^st^ block of Day 1 / 4^th^ block of Day 1 / Day 2). All paired comparisons were subjected to Bonferroni correction. The adult Korean repetition results from a previous experiment [3] were analyzed similarly. Recognition performance for the Recurring items in the *Behavioral experiment* was examined with a t-test on the d’ value.

Group-level statistical analysis of ECD source waveforms in the *MEG experiment* was conducted on the spatiotemporally congruent components that had comparable direction and temporal evolution of current flow. Transient responses were defined by their maximum amplitude and the zero-crossing latency of the declining slope of the responses, determined individually for each participant. For sustained responses the same time windows were used across participants. If significant effects were found in several consecutive time windows, the time windows were pooled together to simplify the description of the results. Recurring stimuli and New stimuli were included in a repeated-measures 2x2x2 ANOVA with the factors Language (Native vs. Foreign), Stimulus type (Recurring vs. New) and Experimental day (Day 1 vs. Day 2). Recurring stimuli refer to the 4^th^ presentations of Recurring words on Day 1 (80), and their single 5^th^ presentation on Day 2 (80); New stimuli refer to all Nonrecurring stimuli on Day 1 (80), presented evenly over the four blocks, and a subset of New stimuli on Day 2 (80). All paired comparisons were subjected to Bonferroni correction.

For evaluating the relationship between behavioral and cortical effects in children in the *MEG experiment*, Spearman’s pairwise correlation was computed between the cortical learning effects and the improvement in repetition accuracy between New and Recurring items during the first experimental session. The neural measure was signal change (percentage) between cortical response to all the New items and the 4^th^ repetition of the Recurring items, normalized to each participant’s average activation level. The improvement in behavioral repetition accuracy was estimated as the difference in repetition accuracy between all the New items and the 4^th^ repetition of the Recurring items, normalized to each participant’s average repetition performance level.

## Results

### Behavioral effects

**Fig 2:**
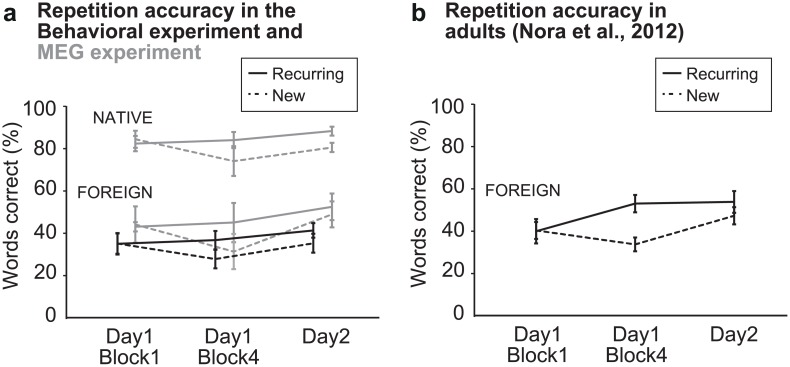
Behavioral learning effects. The Behavioral experiment was otherwise similar to the MEG experiment (Fig 1) except that it included only foreign words as stimuli and stimulus presentation was immediately followed by overt repetition. **(A)** Percentage of correctly repeated Recurring and New words (mean ± SEM) during the 1^st^ and 4^th^ block of Day 1 (both blocks containing 80 Recurring and 20 New words) and Day 2 (in a randomly selected subset of items containing approximately 20 Recurring and 40 New word forms) in children. **(B)** Percentage of correctly repeated Recurring and New words (mean ± SEM) from a similar paradigm in adults [3] for comparison.

The *Behavioral experiment* evaluated repetition and recognition accuracy of foreign (Korean) word forms. Repetition accuracy improved significantly for Recurring word forms [main effect of Stimulus type (Recurring vs. New): F(1,14) = 11.2, p = 0.005; interaction of Stimulus type (Recurring vs. New) and Time of testing (1^st^ block of Day 1 / 4^th^ block of Day 1 / Day 2): F(2,13) = 6.0, p = 0.014] (Fig 2A). In paired comparisons, the difference in repetition accuracy between Recurring and New words was significant in the 4^th^ block of Day 1 and on Day 2 [1^st^ block Day 1: t(14) = 0.05, p = 0.96; 4^th^ block Day 1: t(14) = 4.7, p < 0.001; Day 2: t(14) = 3.3, p = 0.005; Bonferroni corrected alpha = 0.008]. Children correctly recognized 73% of the Recurring Korean word forms in the recognition test at the end of Day 2 of the *Behavioral experiment*. However, the recognition performance only approached significantly better than chance [d’ = 0.68; t(14) = 2.0, p = 0.063].

In the *MEG experiment* learning effects did not reach significance in the available subsample of children when tested separately for each language, but in visual inspection seemed similar to the behavioral experiment in both languages, although overall the performance was higher for the Native language (Fig 2A). Pooling the results of the two language together for statistical analysis confirmed the learning effects found in the *Behavioral experiment*: Improvement in repetition accuracy was seen for Recurring but not for New word forms [interaction of Stimulus type and Time of testing: F(2,8) = 4.9, p = 0.04]. In paired comparisons, the difference in repetition accuracy between Recurring and New words was statistically significant in the 4^th^ block of Day 1 as well as on Day 2 [1^st^ block Day 1: t(14) = 0.47, p = 0.65; 4^th^ block Day 1: t(12) = 3.8, p = 0.002; Day 2: t(11) = 3.8, p = 0.003; Bonferroni corrected alpha = 0.017].

To obtain a reference point of Foreign word repetition accuracy in adults, data obtained in a previous experiment [3] using a similar paradigm in 10 adults were reanalyzed for a qualitative comparison (Fig 2B). The adult results also showed significant improvement in repetition accuracy for Recurring Foreign word forms [main effect of Stimulus type (Recurring vs. New): F(1,7) = 11.6, p = 0.011; interaction of Stimulus type (Recurring vs. New) and Time of testing (1^st^ block of Day 1 / 4^th^ block of Day 1 / Day 2): F(2,6) = 10.6, p = 0.011]. In paired comparisons, the difference in repetition accuracy between Recurring and New words was significant in the 4^th^ block of Day 1 [1^st^ block Day 1: t(7) = 0.13, p = 0.90; 4^th^ block Day 1: t(7) = 5.4, p = 0.001; Day 2: t(7) = 1.4, p = 0.20; Bonferroni corrected alpha = 0.017].

### Brain responses to new word forms

**Fig 3:**
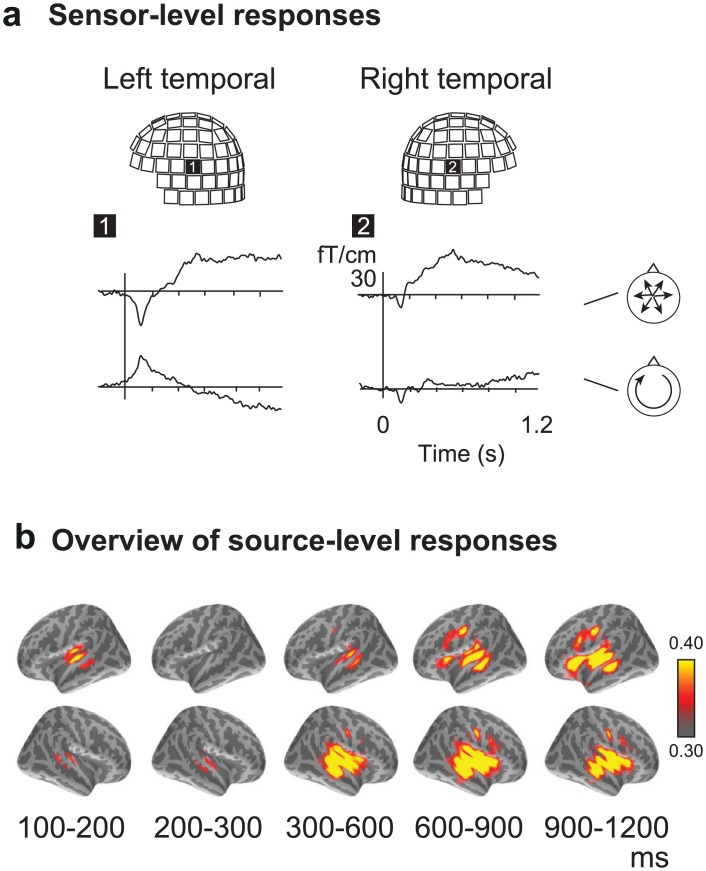
Sensor and source level cortical responses. **(A)** Sensor-level responses, averaged across participants and stimulus conditions. Responses are illustrated at two symmetrical recording sites over the left (site 1) and right (site 2) temporal cortices, with each site containing two planar gradiometers that are sensitive to orthogonal orientations of neural currents (cf. small schematic heads) and detect the strongest signal directly above an active cortical area. **(B)** Noise-normalized Minimum Norm Estimates (dynamical Statistical Parametric Maps, dSPMs) from 100 to 1200 ms after stimulus onset (time slots of 100 ms, then 300 ms), represented as a grand average across participants and conditions, morphed to a standard brain and normalized. Salient activation maxima are evident in the bilateral temporal and left frontal cortices.

The spatiotemporal pattern was comparable for all stimulus categories and across languages, in both the MEG sensor signals and the distributed source-level cortical estimation (Minimum Norm Estimate, MNE). Transient activation was seen bilaterally in the superior temporal cortex, peaking at ~140 ms and returning to baseline at ~300 ms, followed by a strong sustained response from ~300 ms onwards (Fig 3A and 3B). For most of the children, the early transient response showed an inferior-to-superior direction of current flow, whereas an approximately opposite superior-to-inferior direction of current flow was observed in the sustained response. Sustained activation was also evident in the left frontal cortex.

### Early cortical effects

**Fig 4:**
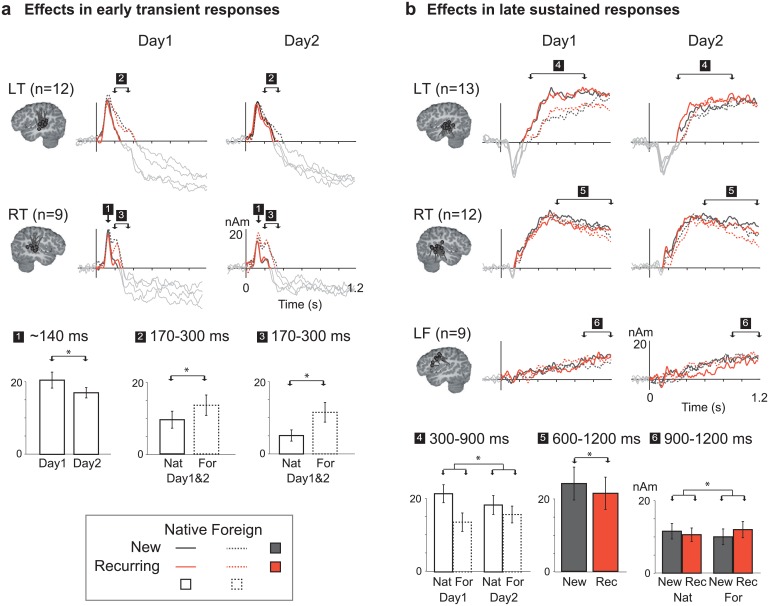
Neural effects. Time courses of activation in sources modeling **(A)** the early transient responses and **(B)** the late sustained responses. Equivalent Current Dipole (ECD) clusters are displayed on a sagittal plane of a standard brain. Each dot represents the center of an active cortical patch in one subject, and the attached line denotes the mean direction of current flow in that area (LT = left temporal, RT = right temporal; LF = left frontal). Grand average source waveforms (activation strength in nanoamperemeters, nAm) are displayed for each ECD cluster on Day 1 and Day 2 (columns). The arrows indicate the time windows that showed statistically significant effects (indexed by numbers 1 through 6; see Table 1 for a full listing of statistical tests). Summaries of significant effects in these time windows are shown as bar graphs (cf. indices on the source waveforms). Error bars indicate the standard error of the mean.

The maximum amplitude of the transient response at ~140 ms in the temporal sources was determined individually for each child in a time window from 100 to 170 ms. The declining slope of the response was described with mean source strength in a time window from 170 to 300 ms and individual zero-crossing latency. The earliest statistically significant effect was that of Experimental day in the right hemisphere [F(1, 8) = 7.6, p = 0.024]: The peak amplitude of the transient activation at ~140 ms was lower on Day 2 [21.9 ± 3.0 nAm, mean ± SEM] than on Day 1 [25.3 ± 2.9 nAm] (Fig 4A, Table 1). In the subsequent time window at 170–300 ms, the bilateral temporal activation was stronger for Foreign than Native stimuli [LH: F(1, 11) = 6.7, p = 0.025, Foreign 13.0 ± 2.7 nAm, Native 9.1 ± 2.3 nAm; RH: F(1, 8) = 16.2, p = 0.004, Foreign 11.3 ± 2.7 nAm, Native 5.0 ± 1.5 nAm], or returned to the base level more slowly [LH: F(1, 11) = 5.3, p = 0.041, zero-crossing latency for Foreign 380 ± 45 ms, for Native 300 ± 26 ms; RH: F(1, 8) = 7.4, p = 0.026, zero-crossing latency for Foreign 370 ± 68 ms, for Native 260 ± 32 ms].

**Table 1:** Group-level statistical analysis results for source amplitude values in each time window and source cluster.

**Time window**	**Source cluster**	**N**	**Effect**	**F**	**df**	**p**
**TRANSIENT RESPONSES**						
~ 140 ms (peak)	LT	12	Stimulus type	.65	1, 11	.44
			Language	0.89	1, 11	.77
			Day	4.8	1, 11	.052
			Interactions		1, 11	n.s.
	RT	9	Stimulus type	0.21	1, 8	.66
			Language	2.8	1, 8	.13
			**Day**	**7.6**	**1, 8**	.024*
			Interactions		1, 8	n.s.
170 ms– 300 ms (mean)	LT	12	Stimulus type	1.6	1, 11	.23
			**Language**	**6.7**	**1, 11**	.025*
			Day	.41	1, 11	.53
			Interactions		1, 11	n.s.
	RT	9	Stimulus type	.54	1, 8	.48
			**Language**	**16.2**	**1, 8**	.004*
			Day	.35	1, 8	.57
			Interactions		1, 8	n.s.
**SUSTAINED RESPONSES**						
300–600 ms (mean)	LT	13	Stimulus type	1.2	1, 12	.30
			**Language**	**11.2**	**1, 12**	.006*
			Day	.24	1, 12	.63
			Interactions		1, 12	n.s.
	RT	12	Stimulus type	3.7	1, 11	.082
			Language	.54	1, 11	.48
			Day	.016	1, 11	.90
			Interactions		1, 11	n.s.
	LF	9	Stimulus type	.15	1, 8	.71
			Language	.14	1, 8	.72
			Day	.34	1, 8	.56
			Interactions			n.s.
600–900 ms (mean)	LT	13	Stimulus type	4.4	1, 12	.058
			Language	4.6	1, 12	.052
			Day	0.92	1, 12	.77
			**Day x Language**	**8.6**	**1, 12**	.013*
	RT	12	**Stimulus type**	**7.6**	**1, 11**	.018*
			Language	.37	1, 11	.56
			Day	.00	1, 11	.99
			Interactions		1, 11	n.s.
	LF	9	Stimulus type	.75	1, 8	.41
			Language	.83	1, 8	.39
			Day	.36	1, 8	.57
			Interactions		1, 8	n.s.
900–1200 ms (mean)	LT	13	Stimulus type	.016	1, 12	.90
			Language	1.9	1, 12	.19
			Day	.62	1, 12	.45
			Interactions		1, 12	n.s.
	RT	12	**Stimulus type**	**17.7**	**1, 11**	.001*
			Language	2.9	1, 11	.12
			Day	.057	1, 11	.82
			Interactions		1, 11	n.s.
	LF	9	Stimulus type	.42	1, 8	.53
			Language	.001	1, 8	.98
			Day	.20	1, 8	.67
			**Stimulus type x Language**	**5.9**	**1, 8**	.042*
**Time window**	**Source cluster**	**N**	**Effect**	**F**	**df**	**p**
**TRANSIENT RESPONSES**						
~ 140 ms (peak)	LT	12	Stimulus type	.65	1, 11	.44
			Language	0.89	1, 11	.77
			Day	4.8	1, 11	.052
			Interactions		1, 11	n.s.
	RT	9	Stimulus type	0.21	1, 8	.66
			Language	2.8	1, 8	.13
			**Day**	**7.6**	**1, 8**	.024*
			Interactions		1, 8	n.s.
170 ms– 300 ms (mean)	LT	12	Stimulus type	1.6	1, 11	.23
			**Language**	**6.7**	**1, 11**	.025*
			Day	.41	1, 11	.53
			Interactions		1, 11	n.s.
	RT	9	Stimulus type	.54	1, 8	.48
			**Language**	**16.2**	**1, 8**	.004*
			Day	.35	1, 8	.57
			Interactions		1, 8	n.s.
**SUSTAINED RESPONSES**						
300–600 ms (mean)	LT	13	Stimulus type	1.2	1, 12	.30
			**Language**	**11.2**	**1, 12**	.006*
			Day	.24	1, 12	.63
			Interactions		1, 12	n.s.
	RT	12	Stimulus type	3.7	1, 11	.082
			Language	.54	1, 11	.48
			Day	.016	1, 11	.90
			Interactions		1, 11	n.s.
	LF	9	Stimulus type	.15	1, 8	.71
			Language	.14	1, 8	.72
			Day	.34	1, 8	.56
			Interactions			n.s.
600–900 ms (mean)	LT	13	Stimulus type	4.4	1, 12	.058
			Language	4.6	1, 12	.052
			Day	0.92	1, 12	.77
			**Day x Language**	**8.6**	**1, 12**	.013*
	RT	12	**Stimulus type**	**7.6**	**1, 11**	.018*
			Language	.37	1, 11	.56
			Day	.00	1, 11	.99
			Interactions		1, 11	n.s.
	LF	9	Stimulus type	.75	1, 8	.41
			Language	.83	1, 8	.39
			Day	.36	1, 8	.57
			Interactions		1, 8	n.s.
900–1200 ms (mean)	LT	13	Stimulus type	.016	1, 12	.90
			Language	1.9	1, 12	.19
			Day	.62	1, 12	.45
			Interactions		1, 12	n.s.
	RT	12	**Stimulus type**	**17.7**	**1, 11**	.001*
			Language	2.9	1, 11	.12
			Day	.057	1, 11	.82
			Interactions		1, 11	n.s.
	LF	9	Stimulus type	.42	1, 8	.53
			Language	.001	1, 8	.98
			Day	.20	1, 8	.67
			**Stimulus type x Language**	**5.9**	**1, 8**	.042*

LT = left temporal cortex, RT = right temporal cortex, LF = left frontal cortex. Statistically significant p-values denoted with asterisk (* <.05).

### Late sustained cortical effects

For the ECD components modeling the sustained temporal and frontal responses, mean activation values were considered in three 300-ms time windows (300–600 ms, 600–900 ms and 900–1200 ms), which captured the onset, plateau and decline of the sustained responses. Activation in the sustained left temporal responses was weaker for Foreign than Native language [main effect of Language at 300–600 ms: F(1, 12) = 11.2, p = 0.006, Foreign 11.5 ± 2.3 nAm, Native 17.5 ± 2.9 nAm] (Fig 4B, Table 1). However, this effect was mainly present during Day 1 [interaction Language x Experimental day at 600–900 ms: F(1, 12) = 8.6, p = 0.013, Day 1: Foreign 16.4 ± 2.8 nAm, Native 24.4 ± 2.2 nAm; Day 2: Foreign 19.6 ± 2.7 nAm, Native 20.5 ± 2.4 nAm]. Activation was reduced for the Native and increased for the Foreign stimuli on Day 2 compared to Day 1; however, in paired comparisons, neither of these effects reached significance after correcting for multiple comparisons [Native on Day 1 vs. Day 2: t(12) = 1.3, p = 0.044; Foreign on Day 1 vs. Day 2: t(12) = 1.8, p = 0.097; Bonferroni corrected alpha = 0.025].

**Fig 5:**
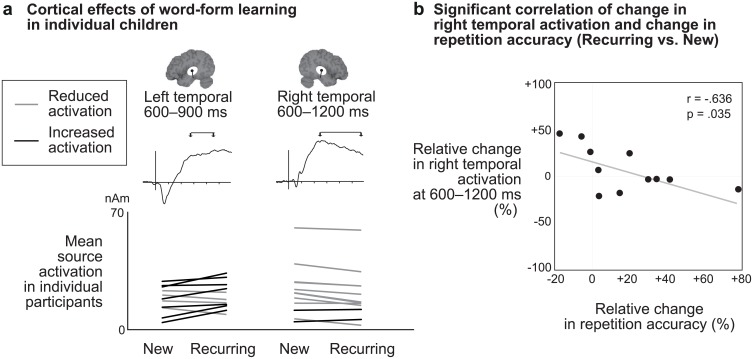
Cortical learning effects in individual children and their significant correlation with behavioral learning effects. **(A)** Effects of word-form level learning in individual children in the sustained left and right temporal activations. Reduced activation to Recurring compared to New word forms was seen in 10/12 children in the right temporal cortex and in 6/13 children in the left temporal cortex. **(B)** Scatterplot of change in right temporal cortical activation as a function of change in repetition accuracy. The y-axis shows the relative (percent) change in the right temporal activation at 600–1200 ms, and the x-axis shows the relative (percent) difference in repetition accuracy, for the 4^th^ presentation of Recurring word forms compared with New word forms.

In the right temporal sustained response, activation was reduced for the Recurring stimuli compared to the New stimuli, similarly for both languages [600–1200 ms: F(1, 11) = 14.7, p = 0.003; Recurring 21.3 ± 4.4 nAm, New 23.9 ± 4.5 nAm]. In the left temporal sustained responses there was a trend for increased responses for Recurring compared to New stimuli [main effect of Stimulus type at 600–900 ms: F(1,12) = 4.4, p = 0.058]. The effects of Stimulus type were more consistent across subjects in the right than left temporal responses (Fig 5A).

In the left frontal sustained response, a significant Stimulus type x Language interaction indicated an increase of activation for the Recurring stimuli compared to the New stimuli for Foreign but not for Native language [900–1200 ms: F(1, 8) = 5.9, p = 0.042; Foreign Recurring 11.2 ± 6.2 nAm, Foreign New 9.3 ± 6.1 nAm; Native Recurring 9.9 ± 5.2 nAm, Native New 10.8 ± 5.9 nAm]. In paired comparisons of Recurring and New stimuli, the effect approached significance only for the Foreign language [900–1200 ms: Foreign: t(8) = 2.0, p = 0.08; Native: t(8) = 0.95, p = 0.37].

### Correlation of behavioral and cortical learning effects

The amount of signal change observed in the right temporal activation at 600–1200 ms from the 1^st^ to the 4^th^ presentation of the Recurring items correlated significantly with the behavioral improvement in repetition accuracy from the 1^st^ to the 4^th^ repetition during Day 1 [ρ_s_ (Spearman’s rho) = 0.65, p = 0.032]: The more the signal strength decreased during learning, the better the participant had become at repeating the Recurring items (Fig 5B). No other significant correlations were found.

## Discussion

We investigated the processing and learning of new foreign and native word forms in school-aged children over two days of exposure. Children showed salient incidental learning of recurring word forms, evidenced as better repetition accuracy for recurring than new words, but they mostly failed in explicit recognition of the recurring items. Comparison to previous repetition data from adults revealed that improvement in repetition accuracy for recurring word forms over two days was similar in adults and children.

Overall, the cortical regions engaged in processing new word forms in children were largely comparable to those in adults, with bilateral superior temporal and left frontal involvement [3, 36]. However, the morphology of the responses and the underlying sources in the temporal regions were different from those previously reported in adults. Early transient responses at ~140 ms in superior temporal areas had a superiorly oriented direction of current flow in most children, confirming earlier results [28], whereas sustained late responses (from ~300 ms onwards) in the bilateral temporal showed an opposite direction of current flow. These sustained responses were similar to adults, but the observed learning effects differed.

### Effects of familiarization to task and language are seen in the right and left superior temporal regions overnight

In superior temporal responses, we observed general changes that could be related to familiarization with the task: The transient activation (at ~140 ms) in the right superior temporal cortex was weaker on day two, akin a similar effect found in adults [3]. Bilateral superior temporal activation differed for foreign and native language, similarly to findings in adults [3, 36]. In the left sustained responses, this language difference was reduced overnight, possibly reflecting increased familiarity of the foreign phonology and in line with previous studies demonstrating that over time the brain starts to respond to a foreign language in a similar manner as to the native language [52–54]. However, brain responses to new native word forms also changed overnight; thus, these general changes might also be related to improvement in the repetition task as a consequence of sleep or a day’s delay.

### Word-form familiarity is reflected in right temporal sustained responses

We observed an effect of word-form-level familiarity as a reduction of right superior temporal activation for the recurring word forms. This effect mirrored the decreased left superior temporal activation found in adults, in both spatial properties and timing [3–6]. Furthermore, the magnitude of decrease in the right temporal activation correlated with the amount of each child's improvement in repetition accuracy, again mirroring our earlier, similar observation for the left-lateralized effect in adults [36]. This suggests that the present right-lateralized effect in children is related to establishing incidental memory representations for the recurring word forms. A trend for increased activation to recurring word forms was observed in the left temporal cortex, but the effects of word form familiarity in this response were quite variable across subjects.

Several studies on normally developing school-aged children have demonstrated bilateral activation or learning effects during language processing and learning in younger children, with a shift to left hemispheric dominance as age increases [9, 32, 55]. The specialization of the left hemisphere for linguistic processing with age was originally thought to result from maturational processes alone, and to underlie children’s superiority in language learning [56], but other possible conceptualizations have been subsequently put forward [57, 58]. The increasing functional specialization of neural systems underlying language processing has been suggested to be driven by proficiency in the given language rather than brain maturation, and has been tightly linked to performance [8, 54, 59]. In most studies, however, the comparison has been conducted between known words and new words of the native language, differing both in terms of lexical word form and meaning familiarity. In the current study, we focused on learning new word forms without referents, which allowed us to address the cortical effects of the new words being phonologically familiar. Crucially, a right-hemispheric effect was observed similarly for recurring foreign and native word forms, implying that right-hemispheric processes are involved in children even when learning new word forms of the native language. Thus, the present results indicate that even as late as in school-age, the left-hemispheric regions that are specialized for language processing may not yet fully dominate the learning of new phonological forms of a language, irrespective of proficiency in the language, whereas in adults left-hemispheric processing is emphasized [3, 36]. Compatibly, prominent bilateral activation has been observed in children for unknown words of the native as well as foreign language [9, 60].

Tentatively, the observed hemispheric differences in the familiarity effects between children and adults could be indicative of a general qualitative shift in the way phonological stimuli are processed [61]. Studies on speech processing in adults and children have suggested that left-hemispheric areas serve phoneme-level processes, while right temporal regions dominate in suprasegmental processing and handling of syllable rate modulations [62–65]. We speculate that the right-lateralized word form learning effect in children could be related to reliance on suprasegmental information such as intonation contours, whereas adults rely largely on detailed and efficient phoneme-level processing in the left hemisphere. This suggestion is compatible with results showing that word form representations in school age continue to be less detailed and more holistic than in adults [26, 66]. Children might focus their attention on melodic and rhythmic aspects of the novel word forms, what has been found to result in more right-lateralized responses [67, 68]. The lack of fine-grained segmental representations for new word forms could also explain why, in a recognition task, children fail to explicitly discriminate between newly-learned and unknown words with overlapping acoustic properties.

Speech comprehension and production can both be thought to be mediated by shared phonological representations that emerge from the mapping of acoustic input to motor output [69]. Less detailed phonological representations for new word forms in children would, therefore, likely lead to a cumbersome translation of these phoneme sequences into articulatory plans, a process reflected in activation of the left premotor areas [36]. This could explain why the frontal responses were generally weak and showed small learning effects in children, whereas in adults a prominent increase in activation for recurring compared to new stimuli has been observed [3, 36].

## Conclusions

The current results reveal a salient right-hemispheric contribution to learning new word forms in children, similarly for native and foreign language. We propose that this might be due to a qualitatively different processing in children compared to adults: in processing new word forms, children rely on suprasegmental prosodic information supported by right-hemispheric processing, and have not yet grown fully constrained to segmenting the input into established native-language phoneme representations in the specialized left temporal areas. It remains for future studies to investigate, possibly over longer periods of exposure, whether the differential cortical learning effects in comparison to adults might reflect differences in language learning potential.
